# Beyond Combustion: Oxidative Stress as a Shared Pathway of Harm Across Conventional Cigarettes, Heated Tobacco Products, E-Cigarettes, and Waterpipe Smoking

**DOI:** 10.3390/antiox15091131

**Published:** 2026-09-07

**Authors:** Lorenzo Loffredo

**Affiliations:** 1 Interdisciplinary Department of Well-Being, Health and Environmental Sustainability (BESSA), Sapienza University of Rome, 02100 Rieti, Italy; lorenzo.loffredo@uniroma1.it; 2 I Clinica Medica, Policlinico Umberto I, Viale del Policlinico 155, 00161 Rome, Italy

## 1. Introduction

Tobacco smoking is an established cardiovascular risk factor and is closely associated with the development and progression of atherosclerosis, as well as complications such as myocardial infarction and stroke [1,2,3]. In recent years, there has been a rapid spread of alternatives to conventional cigarettes—including heated tobacco products (HTPs), e-cigarettes, and water pipes—especially among adolescents and young adults [4,5,6,7]. Such a spread has been favored, at least in part, by the perception that the absence of combustion or the filtration of smoke through the water substantially reduces the health risk compared with conventional cigarettes [8,9].

The available evidence indicates, however, that these products are not free of harmful effects [10,11]. Nicotine, reactive aldehydes, ultrafine particles, volatile organic compounds, and metals present in their aerosols can impair endothelial function, promote inflammation, and contribute to cardiovascular, breathing and systemic damage, even in the absence of combustion [9,10,12]. Consequently, several scientific societies have reiterated that, as well as traditional cigarettes, e-cigarettes, HTPs, and waterpipes should also not be considered safe products and that complete cessation of tobacco and nicotine exposure remains the priority goal, prioritizing counseling and validated drug therapies over their use as harm reduction tools [8,12,13,14,15,16].

Oxidative stress appears to be one of the main mechanisms shared by these different smoking products. Exogenous oxidants and the activation of cellular sources of reactive oxygen species (ROS)—including NADPH oxidases and mitochondria—can reduce antioxidant defenses, impair nitric oxide bioavailability, and trigger proinflammatory responses [10]. Persistence of this redox alteration can result in cellular dysfunction and multiorgan damage [10,17,18]. However, the mechanisms through which different types of exposure induce and maintain oxidative stress and systemic inflammation are not yet fully defined. This Special Issue and this Editorial aim to critically analyze the evidence relating to the effects of conventional cigarettes and new tobacco and nicotine products on redox balance, delving into their potential links with persistent inflammation and consequent organ damage.

## 2. Overview of Published Articles

### 2.1. Effect of Smoking and Increase in Oxidized Stress Markers

#### 2.1.1. Conventional Cigarettes

**Figure 1:**
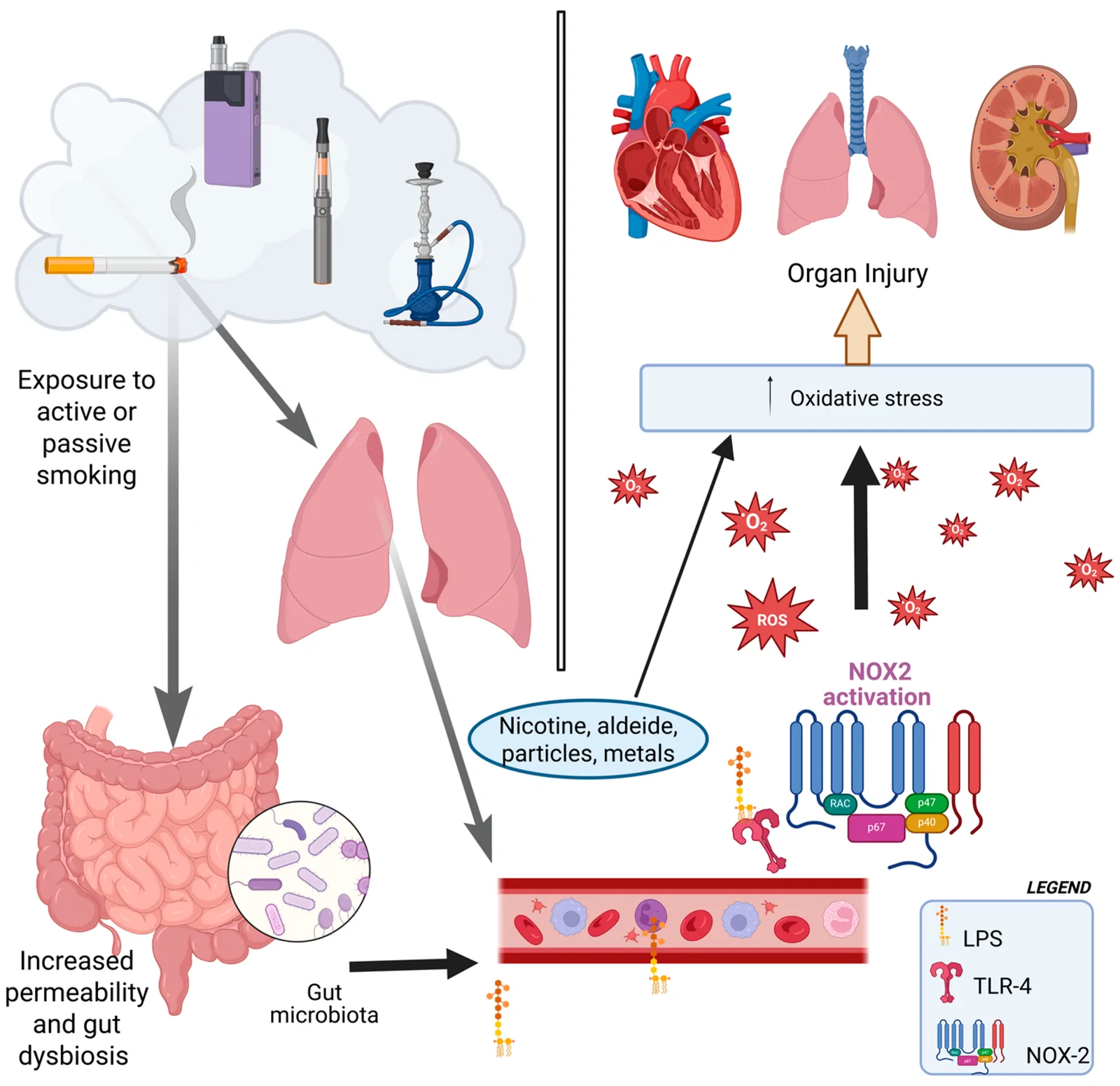
Proposed mechanisms linking exposure to conventional, heated tobacco and electronic smoking products to systemic oxidative stress and organ injury. Active or passive exposure to cigarette smoke, electronic cigarettes, heated tobacco products, and waterpipe tobacco smoke introduces nicotine, reactive aldehydes, particulate matter, and metals into the respiratory tract. These constituents may directly increase reactive oxygen species (ROS) formation and promote NOX activation. Smoking exposure may also increase intestinal permeability and induce gut dysbiosis, facilitating the translocation of bacterial lipopolysaccharide (LPS) into the circulation [Contribution 1]. LPS binding to Toll-like receptor 4 (TLR4), together with smoke-related toxicants, activates intracellular signaling pathways that promote the assembly of the NOX2 complex through the recruitment of Rac and the cytosolic p47phox, p67phox, and p40phox subunits to the membrane-bound catalytic complex. Activated NOX2 generates superoxide, thereby amplifying systemic oxidative stress. The resulting redox imbalance and inflammatory response contribute to cardiovascular, pulmonary, and renal injury.

The damage caused by conventional cigarettes well established [10,11]. As reported in several previous studies, and in the one reported in this Special Issue, the conventional cigarette is associated with a greater increase in oxidative stress markers such as NOX2, isoprostanes, and H_2_O_2_ and a reduction in antioxidant status highlighted by the reduction in NO [10,19,20] [Contribution 1]. This increase occurs in subjects exposed to both secondhand smoke and active smoke. A novelty reported in this Special Issue is that conventional cigarette smoking can cause an alteration of the intestinal microbiota, favoring the passage of lipopolysaccharide (LPS)—a membrane component of Gram-negative bacteria—into the systemic circulation, with subsequent binding to Toll-like receptor 4 (TLR4) and activation of oxidative stress through NADPH oxidase type 2 (NOX2) (Figure 1) [Contribution 1]. This increased intestinal permeability could perpetuate and contribute to the increase in oxidative stress seen in smokers (Figure 1) [Contribution 1]. Of particular interest is the damage found in children exposed to conventional tobacco cigarettes [19] [Contribution 1].

#### 2.1.2. HTPs

Like conventional tobacco cigarettes, HTPs also cause increased oxidative stress and a reduction in antioxidant status [19,21] [Contribution 1]. Of pivotal importance is the lack of significance in blood concentrations of LPS and zonulin between subjects actively and passively exposed to traditional cigarette smoke and those exposed to HTPs, which highlights the detrimental effect of both of these products on the systemic concentration of oxidative stress and NOX2 activation [Contribution 1].

Another interesting in vitro experimental study, conducted on human bronchial epithelial cells exposed for 24 h to emissions from three different HTPs, reports that such emissions can induce mitochondrial oxidative stress [Contribution 2]. Although HNBC resulted in less extensive overall alterations in gene expression than the conventional cigarette, all examined products significantly increased the production of ROS at the mitochondrial level [Contribution 2]. This is in line with the review by Morosini [Contribution 3] and with other previous studies on the damage caused by these products in terms of increased inflammatory and oxidative status [Contribution 3] [22,23].

#### 2.1.3. Electronic Cigarettes

Compared to other devices with more or less homogeneous characteristics, one of the problems with e-cigarettes is their variability among devices in generating aerosol; there are also numerous types (approximately 8000) of liquid flavors available [11].

Despite these variabilities, e-cigarettes produce volatile harmful products such as formaldehyde, acrolein and fine particles [11] and are also not free from pro-oxidant and inflammatory effects [24,25,26]. Thus, e-cigarettes can activate NOX2 and increase oxidative stress, as assessed by the higher levels of isoprostanes with reduced bioavailability of NO and blood levels of Vitamin E [26,27]. Sater’s review shows that e-cigarette aerosols contain numerous potentially pro-oxidant components and that, in humans, vaping has been associated with alterations in antioxidant defenses and early indicators of vascular damage [Contribution 4].

The main alterations observed concern the following:

- The reduction in superoxide dismutase and catalase;
- The alteration of the ratio between reduced and oxidized glutathione;
- The increased activity of NOX-2, a ROS-producing enzyme;
- The increase in malondialdehyde, an indicator of lipid peroxidation;
- The increase in 8-isoprostanes;
- Increased 8-OHdG, an indicator of oxidative DNA damage.

Nicotine appears to play a significant role in the effects of the damage, but the compounds contained in e-cigarette aerosols could also contribute to it.

#### 2.1.4. Waterpipes

In recent years, an increase in the use of waterpipes with tobacco (WTS) has been observed worldwide, especially among young people, both of Arabic origin and belonging to other populations [16,28,29]. The perception, unsupported by scientific evidence, that WTS is less harmful than conventional cigarette smoke—because the smoke is filtered through water—has contributed to the spread of this practice. However, this belief is not supported by studies, which instead highlight the danger of this practice. In fact, WTS sessions last approximately 45 min, with inhalation of several liters of waterpipe smoke, corresponding to approximately 10 times the absorption of CO and particulate matter, 27 times the formaldehyde, and 80 times the lead compared to a single conventional cigarette [28]. The growing popularity of tWTS among younger age groups led the World Health Organization to define it, in its 2015 advisory note, as an emerging public health problem [28].

In the study by Shaukat et al., published in this Special Issue, the concentration of 8-oxoguanine (8-oxoGua) in exhaled breath condensate (EBC) was analyzed before and after a WTS session [Contribution 5]. 8-oxoGua, a product of DNA guanine oxidation, represents a biomarker of oxidative DNA damage mediated by ROS. After exposure to waterpipe smoke, a significant increase in 8-oxoGua levels was found, indicative of an acute increase in oxidative stress and subsequent oxidative DNA damage [Contribution 5].

### 2.2. Effect of Smoking on Oxidative Stress and Organ Damage

#### 2.2.1. Cardiovascular System

The harmful cardiovascular effects of conventional cigarettes have been well established; numerous meta-analyses have demonstrated a close relationship with coronary heart disease, stroke, and sudden death, making tobacco smoking a classic cardiovascular risk factor [30,31].

Although some studies report that HTPs release lower amounts of several toxic substances than conventional cigarettes, the reduction in exposure does not equate to an absence of health risk [10,11]. The available evidence indicates that their emissions continue to promote oxidative stress, inflammation, endothelial dysfunction and other cellular alterations associated with the development of cardiovascular diseases [19,21] [Contribution 1]. HTPs should therefore not be described as safe products; any harm reduction concerns only comparison with the continuation of conventional cigarette smoking and not with complete abstinence from tobacco products [Contribution 3].

A further element of concern relates to passive exposure. Higher concentrations of cotinine, circulating LPS, and zonulin were found in children passively exposed to HTP emissions or conventional cigarette smoke compared to unexposed controls, with no significant differences between the two forms of exposure. Elevated zonulin may suggest increased intestinal permeability, which may promote the translocation of bacterial LPS into the bloodstream and lead to low-grade endotoxemia [32]. LPS was also associated with markers of NOX2-mediated oxidative stress, reduced nitric oxide bioavailability, and increased platelet activation. These alterations could contribute to endothelial dysfunction and, in the long term, to an increased cardiovascular risk. The study did not, however, directly analyze the intestinal microbiota; therefore, it does not allow us to state that secondhand smoke causes dysbiosis, but identifies an impairment of the intestinal barrier compatible with this hypothesis [Contribution 1].

Another possible mechanism of cardiovascular damage is described in the review by Sabath de Oliveira Bernardes et al. [Contribution 6]. Analysis of clinical and experimental studies shows that exposure to conventional cigarettes and e-cigarettes is associated with increased oxidative stress and impaired cardiac autonomic control [Contribution 6]. The latter is highlighted by the reduction in heart rate variability (HRV), the decrease in parasympathetic modulation and the shift in autonomic balance towards a sympathetic predominance [Contribution 6]. Oxidative stress, inflammation, and sympathetic hyperactivation may promote vascular dysfunction, hypertension, arrhythmias, and other cardiovascular events [Contribution 6]. Although the mechanism is biologically plausible, large studies simultaneously measuring oxidative stress and HRV in smokers are lacking; its prognostic relevance will therefore need to be confirmed by larger longitudinal studies [Contribution 6].

E-cigarettes are also associated with cardiovascular complications [11]. Previous studies showed endothelial dysfunction and reduced coronary reserve after their use [26,33].

Waterpipe smoking has also been associated with cardiovascular changes such as increased heart rate and blood pressure [34]. Additionally, increased coronary artery disease was reported in heavy waterpipe smokers (more than 40/50 sessions per year) compared to less frequent smokers [35].

#### 2.2.2. Renal System

The review by Dzięgiel et al. describes a biologically plausible mechanism supported by cellular, animal, and observational studies through which smoking—and nicotine in particular—can contribute to kidney damage [Contribution 7]. Nicotine, by binding to nicotinic receptors present in podocytes and other renal cells, may promote the activation of NADPH oxidase and the consequent production of reactive oxygen species (ROS) [Contribution 7]. Increased oxidative stress may compromise the integrity of podocytes and the glomerular filtration barrier, promoting proteinuria, inflammation, cell apoptosis, and extracellular matrix deposition, leading to progression of renal fibrosis [Contribution 7]. Clinical evidence therefore suggests that, in patients with glomerulopathy or chronic kidney disease, smoking represents a modifiable risk factor potentially associated with a more rapid decline in renal function [Contribution 7]. However, because much of the mechanistic evidence comes from preclinical models, and clinical studies are predominantly observational, larger longitudinal studies are needed to more precisely define the causal relationship and quantify the renal benefits of smoking cessation [Contribution 7].

Since smoking products such as conventional, electronic and heated tobacco cigarettes and waterpipes contain nicotine, it cannot be ruled out that nicotine-mediated mechanisms could determine potential harmful effect on the kidneys [36,37]. However, differences in nicotine delivery, patterns of use, and exposure to other toxicants preclude the assumption of an equivalent degree of renal risk across different tobacco and nicotine products.

#### 2.2.3. Respiratory System

The systematic review by Cinicola et al. evaluated the relationship between passive smoking exposure, oxidative stress, and pediatric respiratory diseases. The authors describe the harmful effects that secondhand smoke causes in children, contributing to increased oxidative stress and persistent inflammation that causes persistent allergic rhinitis, greater wheezing severity, poorer asthma control and reduced responsiveness to corticosteroids [Contribution 8].

In parallel, a higher proportion of active smokers among adolescents has been reported with e-cigarette or vaping-associated lung injury (EVALI) [38,39].

Children and adolescents are increasingly actively and passively exposed to smoke from new devices [10]. It is therefore of fundamental importance to inform parents about the potential harm resulting from secondhand smoke and to promote smoking cessation campaigns among adolescents which lead to a restoration of the early respiratory damage caused by exposure to smoke [40].

## 3. Conclusions

All currently available types of smoking are characterized by increased oxidative stress and systemic inflammation. E-cigarettes, HTPs, and waterpipes are often identified as less harmful products, a perception that may contribute to their increasing use. However, reduced exposure to certain toxicants should not be equated with an absence of health risks, and current evidence does not support the consideration of these products as harmless.

Therefore, it is of fundamental importance to promote preventive campaigns that highlight the possible damage mediated by oxidative stress at the respiratory, cardiovascular and renal levels. Larger prospective studies will be needed to evaluate the long-term systemic effects of these products.
